# Clinical Profile and Therapeutic Challenges in Atrial Fibrillation Patients with Moderate-to-Severe Chronic Kidney Disease: Insights from the CRAFT Registry

**DOI:** 10.3390/jcm15145639

**Published:** 2026-07-18

**Authors:** Katarzyna Złotorzyńska, Janusz Bednarski

**Affiliations:** 1 Department of Emergency and Disaster Medicine, Faculty of Medicine, Lazarski University, 02-662 Warsaw, Poland; 2 Department of Cardiology, St. John Paul II Western Hospital, 05-825 Grodzisk Mazowiecki, Poland; medbed@wp.pl; 3 Clinic of Cardiology, Lazarski University, 02-662 Warsaw, Poland

**Keywords:** atrial fibrillation, comorbidity, anticoagulation, renal insufficiency, chronic

## Abstract

**Background/Objectives**: Atrial fibrillation (AF) and chronic kidney disease (CKD) frequently coexist and are associated with a high risk of cardiovascular complications. Previous studies have primarily focused on differences in clinical profiles by anticoagulant therapy or arrhythmia. Only a limited number of studies have evaluated the impact of CKD severity on patients’ clinical characteristics. **Methods**: This retrospective observational study aimed to characterize the clinical profile of contemporary patients with AF and CKD with an estimated glomerular filtration rate (eGFR) of 15–49 mL/min/1.73 m^2^. The analysis included patients with AF from the CRAFT registry (NCT02987062). Patients were divided into two groups according to eGFR: 15–49 mL/min/1.73 m^2^ and ≥50 mL/min/1.73 m^2^. The groups were compared with respect to demographic characteristics, comorbidities, and treatment patterns. Statistical analyses included the Mann–Whitney U test, the chi-square test and multivariable logistic regression analysis. **Results**: A total of 3203 patients with AF were included, of whom 1153 had eGFR < 50 mL/min/1.73 m^2^. Compared with patients with eGFR ≥ 50 mL/min/1.73 m^2^, those with lower eGFR were significantly older, more often female, and had a higher burden of comorbidities, including arterial hypertension, heart failure, coronary artery disease, and diabetes mellitus. Direct oral anticoagulants were the predominant anticoagulant therapy, irrespective of renal function. **Conclusions**: Patients with AF and moderate-to-severe CKD present a distinct clinical profile characterized by advanced age and a higher burden of comorbidities. These findings improve the understanding of the clinical profile of patients with AF and moderate-to-severe CKD and may support risk assessment and clinical decision-making.

## 1. Introduction

Atrial fibrillation (AF) is the most common arrhythmia in the general population, affecting approximately 3% of individuals over 21 years of age [1].

The prevalence of AF is particularly high among patients with chronic kidney disease (CKD). This may be related to electrolyte disturbances, abnormalities of calcium–phosphate metabolism, and fluid overload, which are especially common in advanced stages of CKD. AF is estimated to occur in 16–21% of patients with CKD, and in the dialysis population, this proportion increases to as much as 40% [2]. The coexistence of AF and CKD is associated with a markedly increased risk of thromboembolic events, bleeding complications, and all-cause mortality [3].

At the same time, lifestyle changes and ongoing societal development have contributed to an increasing prevalence of overweight and obesity, which are closely associated with hypertension and diabetes mellitus. These conditions are major risk factors for both the development and progression of CKD, contributing to the growing number of affected patients.

Most previous studies have focused on patients with AF, stratified by anticoagulant therapy or arrhythmia subtype.

The aim of the present study was to provide a detailed characterization of the clinical profile of patients with AF and to compare two populations: those with an estimated glomerular filtration rate (eGFR) ≥ 50 mL/min/1.73 m^2^ and those with an eGFR of 15–49 mL/min/1.73 m^2^. Identifying differences between these groups may provide new insights into patients with AF and coexisting renal dysfunction.

## 2. Materials and Methods

### 2.1. Study Description

This registry-based study uses data from the Polish CRAFT registry (MultiCenter expeRience in AFib patients Treated with OAC), registered in the ClinicalTrials.gov (NCT02987062). The registry comprises a retrospective analysis of hospital discharge records of patients with AF treated with oral anticoagulants, including vitamin K antagonists (VKAs) and direct oral anticoagulants (DOACs). The diagnosis of atrial fibrillation was established by the treating physicians according to the applicable ESC guidelines in force at the time of hospitalization and documented in the hospital discharge records.

According to the applicable Polish regulations, ethics committee approval and informed consent were not required due to the retrospective design of the study, which was based on hospital discharge records.

Initially, all patients with AF, regardless of age, hospitalized at a metropolitan academic center and a district hospital between 2011 and 2016 were included.

Exclusion criteria included anticoagulant therapy prescribed for indications other than AF, such as venous thromboembolism or a mechanical heart valve.

The registry collected data on demographics, comorbidities, AF type (paroxysmal, persistent, or permanent), selected laboratory parameters, and the treatment at hospital discharge. Since 2017, the CRAFT registry has been conducted exclusively in the district hospital. Although the CRAFT registry was initially conducted in two centers, the present analysis was based exclusively on patients from the district hospital between 2014 and 2022. Therefore, inter-center heterogeneity did not affect the present study.

### 2.2. Statistical Analysis

Statistical analysis was performed to compare clinical characteristics across eGFR groups. eGFR was calculated using the CKD-EPI (Chronic Kidney Disease Epidemiology Collaboration) formula. A total of 3203 patients with AF were included and divided into two groups:

- eGFR ≥ 50 mL/min/1.73 m^2^ (*n* = 2050).
- eGFR of 15–49 mL/min/1.73 m^2^ (*n* = 1153).

Analyses were performed using Statistica software, version 13 (TIBCO Software Inc., Palo Alto, CA, USA).

The distribution of continuous variables was assessed using the Kolmogorov–Smirnov test with Lilliefors correction. Due to non-normal distribution, the Mann–Whitney U test was used for comparisons.

Categorical variables were analyzed using the chi-square test of independence.

For statistically significant results, the strength of association was assessed using the Phi coefficient and Cramér’s V.

A multivariable logistic regression analysis was performed to identify clinical variables independently associated with eGFR category.

Before model construction, relationships between the explanatory variables were assessed to evaluate potential collinearity. The strength of these associations was weak or very weak; therefore, no variable was excluded from the initial model. The initial model included age, sex, atrial fibrillation type, arterial hypertension, diabetes mellitus, heart failure, and coronary artery disease. Categorical variables were coded as follows. For comorbidities, a value of 1 indicated the presence of a given condition and 0 its absence. Sex was coded as 1 for women and 2 for men. The dependent variable was coded as 0 for patients with an eGFR < 50 mL/min/1.73 m^2^ and 1 for those with an eGFR ≥ 50 mL/min/1.73 m^2^. Variables that did not reach statistical significance in the initial model were removed, and the final model was refitted using the remaining predictors. Consequently, atrial fibrillation type was excluded from the final model. The goodness of fit of the final model was assessed using the Hosmer–Lemeshow test. All statistical tests were conducted with an adopted significance level of α = 0.05.

Detailed statistical results are presented in Table 1, and the distribution of clinical variables in Table 2. Due to missing data, the number of patients included varied across analyses. Analyses were performed using complete-case data for each variable, and the number of missing values for each variable is presented in Table 2. The results of the multivariable logistic regression analysis are presented in Table 3.

## 3. Results

**Figure 1:**
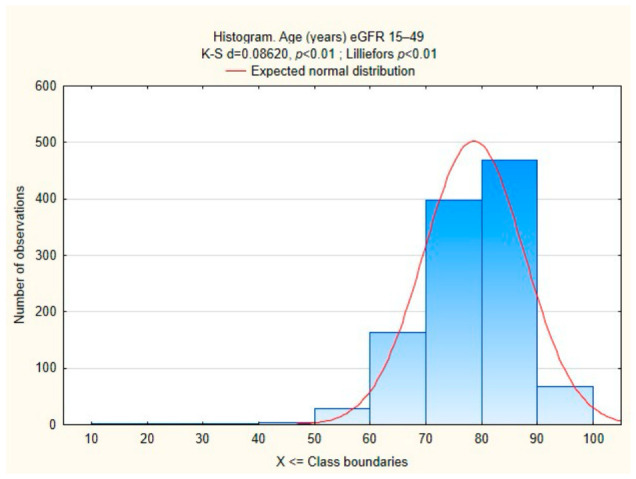
Distribution of age data in the eGFR 15–49 mL/min/1.73 m^2^ group.

**Figure 2:**
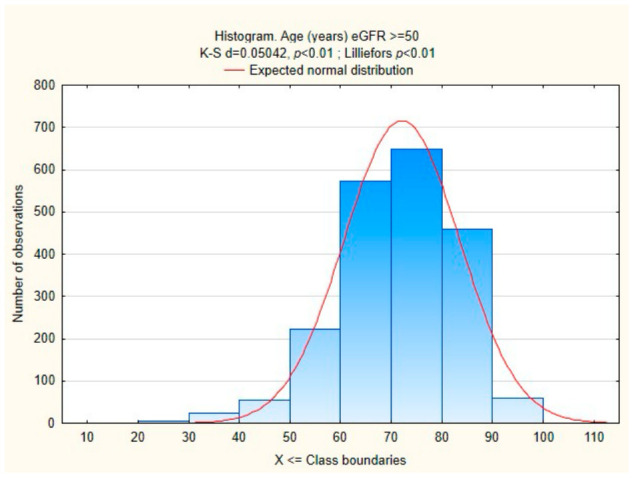
Distribution of age data in the eGFR ≥ 50 mL/min/1.73 m^2^ group.

Patients with eGFR 15–49 mL/min/1.73 m^2^ were older than those with eGFR ≥ 50 mL/min/1.73 m^2^ (mean age 78.6 ± 9 vs. 72.1 ± 11.4 years). The predominant age group was 80–90 years in the lower eGFR group and 70–80 years in the higher eGFR group (Figure 1 and Figure 2).

**Figure 3:**
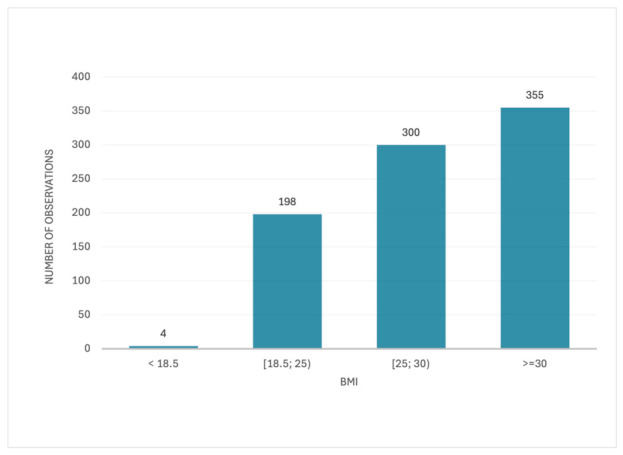
Distribution of BMI data in the eGFR 15–49 mL/min/1.73 m^2^ group.

**Figure 4:**
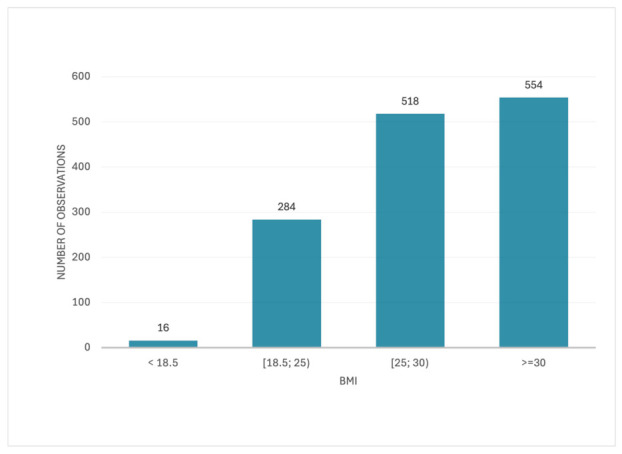
Distribution of BMI data in the eGFR ≥ 50 mL/min/1.73 m^2^ group.

BMI (Body Mass Index) distribution was similar in both groups, with the majority of patients being overweight or obese (Figure 3 and Figure 4).

Women predominated in the eGFR 15–49 mL/min/1.73 m^2^ group (57%), whereas men were more frequent in the eGFR ≥ 50 mL/min/1.73 m^2^ group (61%). Permanent AF was the most common arrhythmia subtype (~50%), followed by paroxysmal AF (~40%) and persistent AF (~10%). Comorbidities—including hypertension, diabetes mellitus, heart failure, and coronary artery disease—were more prevalent in patients with lower eGFR, with the largest difference observed for heart failure (78% vs. 60%). Hyperthyroidism and alcohol or drug dependence were rare and occurred at similar rates in both groups.

Patients with eGFR 15–49 mL/min/1.73 m^2^ had a higher mean CHA_2_DS_2_-VASc score than those with eGFR ≥ 50 mL/min/1.73 m^2^ (4.86 vs. 3.79). Among all patients, 82.2% received anticoagulant therapy. DOACs were the predominant treatment (81%) regardless of eGFR, while VKAs were used in 19% of patients.

Multivariable logistic regression analysis demonstrated that older age, female sex, and the presence of arterial hypertension, diabetes mellitus, heart failure, and coronary artery disease were independently associated with a higher likelihood of belonging to the group with reduced renal function (eGFR < 50 mL/min/1.73 m^2^) (Table 3). The final model showed good fit according to the Hosmer–Lemeshow goodness-of-fit test (*p* = 0.485).

## 4. Discussion

The aim of this study was to characterize the clinical profile of patients with AF and concomitant CKD with an eGFR of 15–49 mL/min/1.73 m^2^ and to identify potential differences compared with patients with preserved or mildly impaired renal function.

The eGFR cut-off of 50 mL/min/1.73 m^2^ was adopted in line with major randomized trials, such as ROCKET-AF and RE-LY, in which this threshold is used to determine dose reduction for rivaroxaban and dabigatran [4,5].

Patients with reduced eGFR represent a distinct high-risk population, primarily due to their advanced age and a greater burden of comorbidities.

Several previous studies and large AF registries have evaluated patients according to renal function. However, these analyses primarily focused on anticoagulant treatment strategies, treatment patterns, or clinical outcomes, including mortality, thromboembolic events, and bleeding complications. The HERA-FIB registry is one of the few studies presenting baseline clinical characteristics according to eGFR categories [6]. The primary objective of the present study was to provide a detailed clinical characterization of patients with AF and moderate-to-severe CKD in comparison with those with preserved or mildly impaired renal function in a contemporary real-world cohort, thereby complementing the existing evidence on patients with AF and CKD.

Across AF registries, patients aged 65 years and older predominate. In the study by Salbach et al., patients with impaired renal function were significantly older than those with normal renal function [6]. A similar relationship was observed in our analysis, where patients with eGFR of 15–49 mL/min/1.73 m^2^ were on average 6.5 years older.

This association likely reflects the higher burden of comorbidities in older individuals, particularly hypertension and diabetes mellitus, which are key risk factors for CKD development and progression. Renal function typically declines progressively with age, at a rate of approximately 1–2 mL/min/1.73 m^2^ per year, further explaining lower eGFR values in older patients. Moreover, advanced age itself is associated with faster CKD progression.

Overweight and obesity predominated in our study population, consistent with global trends observed in AF cohorts [7,8].

In line with previous studies, men predominated in the group of patients with eGFR ≥ 50 mL/min/1.73 m^2^ [9,10]. However, women were more frequent in the group with eGFR 15–49 mL/min/1.73 m^2^. A similar trend has been reported in an Italian study of patients with stage G4 CKD [11]. In the HERA-FIB registry, men still constituted the majority among patients with reduced eGFR, although the proportion was more balanced [6].

Although male sex is generally considered a risk factor for both AF and CKD, the increasing proportion of women observed in advanced CKD stages may indicate that sex-related differences become less pronounced with disease progression. Given the limited available data, these findings should be interpreted cautiously but support the need for further studies evaluating sex-specific differences across CKD stages.

Permanent AF was the most common arrhythmia subtype in our study, which is consistent with findings from the RECALL and the J-RYTHM registries [12,13]. In contrast, other registries, including GLORIA-AF, POL-AF, and PAFF, reported a predominance of paroxysmal AF [14,15,16].

These discrepancies may reflect differences in patient recruitment, particularly between inpatient and outpatient settings. In addition, the predominance of academic centers in certain registries may influence the distribution of AF types. Previous analyses from the CRAFT registry also demonstrated that patients treated in district hospitals were older, more often female, had a higher comorbidity burden, and more frequently presented with persistent AF compared with those treated in academic centers [17].

Heart failure and arterial hypertension were the most prevalent comorbidities among patients with eGFR 15–49 mL/min/1.73 m^2^ (78% and 73%, respectively). These findings are consistent with previous registries, including POL-AF and studies involving patients with advanced CKD [11,15].

Hypertension is consistently reported as the most common comorbidity in AF populations, often exceeding 70% prevalence [8,18,19]. In the HERA-FIB registry, hypertension was present in over 90% of patients with reduced eGFR [6].

The high prevalence of both hypertension and heart failure reflects their bidirectional relationship with CKD, where each condition contributes to the development and progression of the others.

The prevalence of diabetes mellitus in AF populations typically ranges from 12.5% to 33.6%. In our study, diabetes was present in 41% of patients with eGFR 15–49 mL/min/1.73 m^2^, which is among the highest reported values. A similar pattern was observed in the HERA-FIB registry [6].

This finding may be explained by the bidirectional relationship between diabetes and CKD. Chronic hyperglycemia contributes to renal damage, while CKD may exacerbate insulin resistance and impair glucose metabolism.

Coronary artery disease (CAD) followed a similar pattern, with a higher prevalence in patients with reduced eGFR. A similar relationship was observed in the HERA-FIB registry, where CAD prevalence increased with declining renal function [6]. CKD promotes CAD development through mechanisms such as accelerated atherosclerosis, fluid overload, anemia, and metabolic disturbances. In contrast, lower CAD prevalence reported in Japanese and French registries may reflect differences in the studied populations and healthcare systems [20,21,22].

These findings were also associated with a higher mean CHA_2_DS_2_-VASc score in patients with impaired renal function.

In our study, hyperthyroidism occurred at a similar frequency regardless of eGFR level and was more common than in other AF registries, including J-RHYTHM and EORP-AF [13,23]. Although amiodarone use may influence the prevalence of thyroid dysfunction, the higher rates observed in our population are more likely attributable to environmental factors. This observation may be related to the historical iodine deficiency in the Polish population, which has been associated with the development of autonomous thyroid nodules and toxic nodular goiter in older individuals.

Alcohol and drug dependence were relatively uncommon. One possible explanation is the more frequent healthcare contact among patients with advanced CKD, which may encourage better adherence to medical recommendations and healthier lifestyle behaviors.

Regarding anticoagulation, DOACs were the predominant therapy (81%) regardless of renal function. This is consistent with contemporary trends and reflects increasing evidence supporting their efficacy and safety, as well as guideline recommendations [24,25,26].

Compared with earlier registries [18,27,28] where VKAs predominated, the high use of DOACs in our study likely reflects temporal trends and local clinical practice.

This study has several limitations inherent to observational research. The analysis is based on registry data and may be affected by incomplete documentation. Due to its observational design, causal relationships cannot be established. Despite these limitations, the study provides valuable real-world insights into the clinical profile of patients with AF and reduced eGFR.

## 5. Conclusions

This analysis identified significant differences in clinical characteristics between patients with AF and eGFR 15–49 mL/min/1.73 m^2^ and those with eGFR ≥ 50 mL/min/1.73 m^2^.

A typical patient with AF and moderate-to-severe CKD in this cohort was an elderly woman, often overweight or obese, with permanent AF and a high burden of comorbidities, including heart failure, hypertension, diabetes mellitus, and coronary artery disease.

Hyperthyroidism and substance dependence were relatively uncommon. DOACs were the preferred anticoagulant therapy and were used in most patients.

As this descriptive study did not evaluate anticoagulant dosing or clinical outcomes, no conclusions can be drawn regarding the safety or effectiveness of anticoagulant therapy. Further multicenter studies are warranted to better define the characteristics and optimal management of patients with AF and moderate-to-severe CKD.

## Figures and Tables

**Table 1:** Association between eGFR category and clinical variables.

Variable	Test, Statistic	df	*p*-Value	Effect Size (Φ/V)
**Age**	U = 754,329.5	—	*p* < 0.001	—
**Sex**	χ^2^ = 94.42	1	*p* < 0.001	Φ = 0.17
**Type of AF**	χ^2^ = 10.70	2	*p* = 0.005	V = 0.06
**Diabetes mellitus**	χ^2^ = 31.18	1	*p* < 0.001	Φ = 0.10
**Hypertension**	χ^2^ = 12.75	1	*p* < 0.001	Φ = 0.06
**Coronary artery disease**	χ^2^ = 11.90	1	*p* < 0.001	Φ = 0.10
**Alcohol or drug dependence**	χ^2^ = 10.70	1	*p* = 0.001	Φ = 0.10
**Heart failure**	χ^2^ = 107.83	1	*p* < 0.001	Φ = 0.20
**BMI**	χ^2^ = 5.52	3	*p* = 0.137	—
**Hyperthyroidism**	χ^2^ = 0.16	1	*p* = 0.689	—
**Anticoagulant type**	χ^2^ = 0.01	1	*p* = 0.81	—

Φ—phi coefficient, V—Cramer’s V, U—Mann–Whitney statistic, χ^2^—chi-square test. (Abbreviations: AF—Atrial Fibrillation; BMI—Body Mass Index).

**Table 2:** Baseline characteristics of patients.

	eGFR 15–49 mL/min/1.73 m^2^ (*n* = 1153)	eGFR ≥ 50 mL/min/1.73 m^2^ (*n* = 2050)	Missing Values (n)
**Age (mean)**	78.63	72.12	25
**BMI, kg/m^2^** **(mean)**	28.79	28.65	992
**Female sex**	650 (57.27%)	806 (39.36%)	20
Type of AF			
**Paroxysmal**	467 (41.18%)	873 (42.67%)	23
**Persistent**	103 (9.08%)	252 (12.32%)	
**Permanent**	564 (49.74%)	921 (45.01%)	
**Arterial hypertension**	827 (72.86%)	1367 (66.75%)	20
**Diabetes mellitus**	465 (41.01%)	638 (31.17%)	22
**Heart failure**	887 (78.15%)	1229 (60.01%)	20
**Coronary artery disease**	431 (37.97%)	654 (31.93%)	20
**Hyperthyroidism**	116 (10.24%)	219 (10.69%)	22
**Alcohol/drug dependence**	16 (1.45%)	68 (3.46%)	129
**CHA** **_2_** **DS** **_2_** **-VASc score (mean)**	4.86	3.79	None *
Anticoagulant therapy			None **
**VKA**	176 (18.59%)	320 (18.97%)	
**DOAC**	771 (81.41%)	1367 (81.03%)	

* For calculation of the CHA_2_DS_2_-VASc score, missing values for individual components were considered as an indicator of the absence of the respective risk factor, while missing age values contributed 0 points to the age component. ** Missing values refer to unavailable data for a given variable. Patients not receiving oral anticoagulant therapy were not considered missing observations. (Abbreviations: AF—Atrial Fibrillation, BMI—Body Mass Index, DOAC—Direct Oral Anticoagulant, eGFR—estimated Glomerular Filtration Rate, VKA—Vitamin K Antagonist).

**Table 3:** Results of the multivariable logistic regression analysis.

Variable	Reference Category	OR	95% CI	*p*-Value
**Age (years)**	–	1.059	1.050–1.069	<0.001
**Sex**	Female	1.683	1.430–1.981	<0.001
**Hypertension**	Absent	0.830	0.699–0.987	0.035
**Diabetes mellitus**	Absent	0.697	0.591–0.821	<0.001
**Heart failure**	Absent	0.437	0.366–0.521	<0.001
**Coronary artery disease**	Absent	0.783	0.663–0.924	0.004

(Abbreviations: CI—confidence interval; OR—odds ratio).

## Data Availability

The data presented in this study are available on reasonable request from the corresponding author.

## Abbreviations

The following abbreviations are used in this manuscript:

AF	Atrial Fibrillation
CKD	Chronic Kidney Disease
eGFR	Estimated Glomerular Filtration Rate
VKA	Vitamin K Antagonist
DOAC	Direct Oral Anticoagulants
CKD-EPI	Chronic Kidney Disease Epidemiology Collaboration
BMI	Body Mass Index
CAD	Coronary Artery Disease

## References

[1] Haim M., Hoshen M., Reges O., Rabi Y., Balicer R., Leibowitz M. (2015). Prospective National Study of the Prevalence, Incidence, Management and Outcome of a Large Contemporary Cohort of Patients With Incident Non-Valvular Atrial Fibrillation. J. Am. Heart Assoc..

[2] Wanner C., Herzog C.A., Turakhia M.P., Blankestijn P.J., Carrero J.-J., Clase C.M., Deo R., Kasner S.E., Passman R.S., Pecoits-Filho R. (2018). Chronic kidney disease and arrhythmias: Highlights from a Kidney Disease: Improving Global Outcomes (KDIGO) Controversies Conference. Kidney Int..

[3] Olesen J.B., Lip G.Y.H., Kamper A.-L., Hommel K., Køber L., Lane D.A., Lindhardsen J., Gislason G.H., Torp-Pedersen C. (2012). Stroke and Bleeding in Atrial Fibrillation with Chronic Kidney Disease. N. Engl. J. Med..

[4] Patel M.R., Mahaffey K.W., Garg J., Pan G., Singer D.E., Hacke W., Breithardt G., Halperin J.L., Hankey G.J., Piccini J.P. (2011). Rivaroxaban versus Warfarin in Nonvalvular Atrial Fibrillation. N. Engl. J. Med..

[5] Connolly S.J., Ezekowitz M.D., Yusuf S., Eikelboom J., Oldgren J., Parekh A., Pogue J., Reilly P.A., Themeles E., Varrone J. (2009). Dabigatran versus Warfarin in Patients with Atrial Fibrillation. N. Engl. J. Med..

[6] Salbach C., Milles B.R., Hund H., Biener M., Mueller-Hennessen M., Frey N., Katus H., Giannitsis E., Yildirim M. (2024). Effect of impaired kidney function on outcomes and treatment effects of oral anticoagulant regimes in patients with atrial fibrillation in a real-world registry. PLoS ONE.

[7] Steinberg B.A., Gao H., Shrader P., Pieper K., Thomas L., Camm A.J., Ezekowitz M.D., Fonarow G.C., Gersh B.J., Goldhaber S. (2017). International trends in clinical characteristics and oral anticoagulation treatment for patients with atrial fibrillation: Results from the GARFIELD-AF, ORBIT-AF I, and ORBIT-AF II registries. Am. Heart J..

[8] Potpara T.S., Trendafilova E., Dan G.-A., Goda A., Kusljugic Z., Manola S., Music L., Gjini V., Pojskic B., Popescu M.I. (2017). The Patterns of Non-vitamin K Antagonist Oral Anticoagulants (NOACs) Use in Patients with Atrial Fibrillation in Seven Balkan Countries: A Report from the BALKAN-AF Survey. Adv. Ther..

[9] Staerk L., Fosbøl E.L., Gadsbøll K., Sindet-Pedersen C., Pallisgaard J.L., Lamberts M., Lip G.Y.H., Torp-Pedersen C., Gislason G.H., Olesen J.B. (2016). Non-vitamin K antagonist oral anticoagulation usage according to age among patients with atrial fibrillation: Temporal trends 2011–2015 in Denmark. Sci. Rep..

[10] Rutherford O.C.W., Jonasson C., Ghanima W., Söderdahl F., Halvorsen S. (2020). Comparison of dabigatran, rivaroxaban, and apixaban for effectiveness and safety in atrial fibrillation: A nationwide cohort study. Eur. Hear. J.-Cardiovasc. Pharmacother..

[11] Talerico R., Brando E., Luzi L., Vedovati M.C., Giustozzi M., Verso M., Di Gennaro L., Basso M., Ferretti A., Porfidia A. (2024). Safety and effectiveness of oral anticoagulants in patients with atrial fibrillation and stage 4 chronic kidney disease: A real-world experience. Intern. Emerg. Med..

[12] Lopes R.D., de Barros e Silva P.G.M., Filho C.R.H., Cavalvante M.A., Miranda C.M., Esper R.B., de Lima G.G., Ritt L.E.F., da Silva R.M.F.L., Nakazone M.A. (2023). The First Brazilian Cardiovascular Registry of Atrial Fibrillation: Primary Results of the RECALL Study. Am. Heart J..

[13] Atarashi H., Inoue H., Okumura K., Yamashita T., Kumagai N., Origasa H. (2011). The J-RHYTHM Registry Investigators Present Status of Anticoagulation Treatment in Japanese Patients With Atrial Fibrillation—A Report From the J-RHYTHM Registry. Circ. J..

[14] Kozieł M., Teutsch C., Halperin J.L., Rothman K.J., Diener H.-C., Ma C.-S., Marler S., Lu S., Gurusamy V.K., Huisman M.V. (2021). Atrial fibrillation and comorbidities: Clinical characteristics and antithrombotic treatment in GLORIA-AF. PLoS ONE.

[15] Gorczyca I., Jelonek O., Uziębło-Życzkowska B., Chrapek M., Maciorowska M., Wójcik M., Błaszczyk R., Kapłon-Cieślicka A., Gawałko M., Budnik M. (2020). Trends in the Prescription of Non-Vitamin K Antagonist Oral Anticoagulants for Atrial Fibrillation: Results of the Polish Atrial Fibrillation (POL-AF) Registry. J. Clin. Med..

[16] Guenoun M., Cohen S., Villaceque M., Sharareh A., Schwartz J., Hoffman O., Dib J.-C., Ouazana L., Assouline S., Parrens E. (2023). Characteristics of patients with atrial fibrillation treated with direct oral anticoagulants and new insights into inappropriate dosing: Results from the French National Prospective Registry: PAFF. EP Eur..

[17] Bednarski J., Balsam P., Tymińska A., Ozierański K., Żukowska K., Zaleska M., Szepietowska K., Maciejewski K., Peller M., Praska-Oginska A. (2018). District versus academic hospitals: Differences in the clinical characteristics of patients with atrial fibrillation without valvular heart disease treated with oral anticoagulants. Pol. Arch. Intern. Med..

[18] Gorczyca-Michta I., Wożakowska-Kapłon B. (2015). New oral anticoagulants for the prevention of thromboembolic complications in atrial fibrillation: A single centre experience. Kardiol. Pol..

[19] Ha J.T., Scaria A., Andrade J., Badve S.V., Birks P., Bota S.E., Campain A., Djurdjev O., Garg A.X., Harel Z. (2023). Safety and Effectiveness of Rivaroxaban Versus Warfarin Across GFR Levels in Atrial Fibrillation: A Population-Based Study in Australia and Canada. Kidney Med..

[20] Yoshida H. (2021). Is the Japan Diet Instrumental in Preventing Cardiovascular Diseases?. J. Atheroscler. Thromb..

[21] Iqbal I., Wilairatana P., Saqib F., Nasir B., Wahid M., Latif M.F., Iqbal A., Naz R., Mubarak M.S. (2023). Plant Polyphenols and Their Potential Benefits on Cardiovascular Health: A Review. Molecules.

[22] Ruidavets J.B., Ducimetiere P., Evans A., Montaye M., Haas B., Bingham A., Yarnell J., Amouyel P., Arveiler D., Kee F. (2010). Patterns of alcohol consumption and ischaemic heart disease in culturally divergent countries: The Prospective Epidemiological Study of Myocardial Infarction (PRIME). BMJ.

[23] Lip G.Y.H., Laroche C., Dan G.A., Santini M., Kalarus Z., Rasmussen L.H., Oliveira M.M., Mairesse G., Crijns H.J., Simantirakis E. (2013). A prospective survey in European Society of Cardiology member countries of atrial fibrillation management: Baseline results of EURObservational Research Programme Atrial Fibrillation (EORP-AF) Pilot General Registry. EP Eur..

[24] Hindricks G., Potpara T., Dagres N., Arbelo E., Bax J.J., Blomström-Lundqvist C., Boriani G., Castella M., Dan G.A., Dilaveris P.E. (2021). 2020 ESC Guidelines for the diagnosis and management of atrial fibrillation developed in collaboration with the European Association for Cardio-Thoracic Surgery (EACTS): The Task Force for the diagnosis and management of atrial fibrillation of the European Society of Cardiology (ESC) Developed with the special contribution of the European Heart Rhythm Association (EHRA) of the ESC. Eur. Heart J..

[25] Steffel J., Collins R., Antz M., Cornu P., Desteghe L., Haeusler K.G., Oldgren J., Reinecke H., Roldan-Schilling V., Rowell N. (2021). 2021 European Heart Rhythm Association Practical Guide on the Use of Non-Vitamin K Antagonist Oral Anticoagulants in Patients with Atrial Fibrillation. EP Eur..

[26] Joglar J.A., Chung M.K., Armbruster A.L., Benjamin E.J., Chyou J.Y., Cronin E.M., Deswal A., Eckhardt L.L., Goldberger Z.D., Gopinathannair R. (2024). 2023 ACC/AHA/ACCP/HRS Guideline for the Diagnosis and Management of Atrial Fibrillation: A Report of the American College of Cardiology/American Heart Association Joint Committee on Clinical Practice Guidelines. Circulation.

[27] Akao M., Chun Y.H., Wada H., Esato M., Hashimoto T., Abe M., Hasegawa K., Tsuji H., Furuke K. (2013). Current status of clinical background of patients with atrial fibrillation in a community-based survey: The Fushimi AF Registry. J. Cardiol..

[28] Boriani G., Laroche C., Diemberger I., Fantecchi E., Popescu M.I., Rasmussen L.H., Dan G.-A., Kalarus Z., Tavazzi L., Maggioni A.P. (2016). ‘Real-world’ management and outcomes of patients with paroxysmal vs. non-paroxysmal atrial fibrillation in Europe: The EURObservational Research Programme–Atrial Fibrillation (EORP-AF) General Pilot Registry. Europace.

